# Read-the-game: System for skill-based visual exploratory activity assessment with a full body virtual reality soccer simulation

**DOI:** 10.1371/journal.pone.0230042

**Published:** 2020-03-17

**Authors:** César Daniel Rojas Ferrer, Hidehiko Shishido, Itaru Kitahara, Yoshinari Kameda

**Affiliations:** 1 Ph.D. Program in Empowerment Informatics, School of Integrative and Global Majors (SIGMA), University of Tsukuba, Tsukuba, Japan; 2 Center for Computational Sciences (CCS), University of Tsukuba, Tsukuba, Japan; Middlesex University, UNITED KINGDOM

## Abstract

We present a novel virtual reality (VR) system to measure soccer players’ read-the-game ability. Read-the-game is a term that encompasses a conglomerate of visual exploratory behavioral patterns and cognitive elements required to make accurate in-game decisions. Our technological approach in the Sports Science domain focuses on the visuomotor component of targeted skill development in a VR simulation because VR is a powerful perception–action coupling training solution for visuomotor coordination due to its high sense of immersion and its psychological byproduct presence. Additionally, we analyze two critical aspects: psychological (i.e., sense of presence) and the human–computer interaction (HCI) domain (i.e., suitable input device for full-body immersion). To measure head movements related to visual explorations, the system tracks the user’s head excursions. Specifically, the engaged visual exploratory activity (VEA) during a VR simulation is measured frame-by-frame at runtime to study the behavior of players making passing decisions while experiencing pressure from rivals during in-game situations recreated with computer graphics (CG). Additionally, the sense of presence elicited by our system is measured via the Igroup Presence Questionnaire applied to beginner and amateur soccer players (n = 24). Regarding the HCI aspect, a comparison of input options reveals that a high presence can be achieved when using full body interactions that integrate head and body motions via a combination of an HMD and kinetic body tracking. During our system verification, a difference in the VEA performance is observed between beginner and amateur players. Moreover, we demonstrate the capacity of the system to measure the VEA while evoking immersive soccer in-match experiences with a portable VR setup.

## Introduction

In association football (soccer), an essential element of the game is passing the ball to a teammate who is in the most favorable position to receive it while simultaneously tracking of the locations of rival players. Elite players quickly make accurate decisions based on visual information while considering another player’s dominant region, which is the area where a person can be the first to arrive [1]. The term employed to encompass the conglomerate of visual exploratory abilities needed to make the most accurate in-game decisions and react to unfolding events is referred to as the read-the-game skill [2]. From a sports science perspective, skilled perception is critical for good in-game decision-making and excellent performance [3].

Previous research suggests that a higher frequency of head movements translates into better ball performance [4]. Furthermore, an increased visual exploratory activity (VEA) is linked with a higher probability of performing a successful pass [5]. Related research involving a video projection screen supports these assertions as experienced players fixate in more locations in the periphery and players positions before receiving a pass, while inexperienced ones tend to fixate on the ball and the player with the ball [6]. Although there is a consensus among coaches about the importance of visually scanning the surroundings before making a pass [7], technological alternatives to standard soccer pitch training drills that present body action requirements with a significant perceptual stimulus to the player are scarce.

Other researchers have pondered the need to validate alternative technologies to investigate eye, head, and body movements related to VEA of soccer players engaged in typical open-play situations [8]. One work demonstrated the potential of training solutions that encourage frequent VEA to promote successful performance [9]. Herein we explore a new method to measure and train read-the-game ability before performing a pass while promoting frequent visual explorations.

Due to the complexity of the targeted skill, one critical component related to this ability is examined. Specifically, the targeted behavior is VEA (i.e., how much an individual actively rotates the head and body in search of visual information) [10].

To acquire head tracking data, we use virtual reality (VR) and a commercially available head mounted display (HMD). Inside the VR simulation, soccer players make passing decisions based on continuous visual explorations spanning their field of regard (FoR) and react accordingly under pressure from rivals. In other words, the rotation of the user’s head and body is a significant element of interest in cognitive processes related to accurate in-game passing and decision-making. The HMD rotational data is obtained frame-by-frame during the simulation, whereas FoR is divided into three zones of interest. Consequently, HMD rotational data is obtained as a quantifiable data representation of the VEA performance.

To create a sense of immersion, a cohesive simulation of a real soccer in-game situation is recreated inside a virtual environment (VE) using computer graphics (CG). A realistic spatial representation and audio obtained from ambient sound recordings in real soccer games are used. In the experiment, the player is confronted with a scene in which pressure from a rival player is present in order to establish a sense of realism and to simulate passing decision-making similar to a stressful real-life game scenario.

During the design stage, we considered both system portability and economic feasibility. Both soccer players and managers should benefit from a VR training system that can be deployed in various locations (e.g., a locker room before a match). Hence, using available and portable hardware is important.

Moreover, to realize an immersive VEA training system, we considered an important element from the psychology domain based on our previous research [11]. In general, a high sense of immersion and its psychological byproduct “presence” are essential in VR technology [12]. To measure the level of immersion and more specifically, the subjective sensation of being inside VE, we used the sense of presence as the primary indicator.

For the consecution of a stronger sense of presence, human–computer interaction (HCI) elements are critical. The type of input device impacts the full-body immersive experience. It has been suggested that “one area where virtual reality can add significant value is in understanding what optical variables are important in guiding action” [12]. A strong sense of presence depends on the perception–action coupling. It is achieved in a VR context only when the user can move freely inside VE as if he were in the real world [13]. Hence, the perceptual effect of different input options is analyzed in the VEA performance inside a VR-mediated world to optimize the system design. We analyze two controller options: Kinect and Gamepad. We compare the user IPQ and VEA correlation data with that obtained in our previous study, which used an Xbox controller as the input device [11]. Similar to a previous study for virtual media [14], we use a Microsoft Kinect controller to achieve full body immersion due to its high interactivity.

Towards the realization of an economic and portable read-the-game training system, this study experimentally evaluates the capacity of our proposed system to measure the VEA performance of soccer players while simultaneously providing a high sense of presence. An experiment involving beginner and amateur soccer players (n = 24) is used to test the efficacy. A direct comparison between groups explores potential differences in the VEA scores by player level.

To measure the level of immersion that maximizes the VEA training experience in a VR environment (i.e., provides a high sense of presence), we apply the Igroup Presence Questionnaire (IPQ) post-simulation [15].

### Main contribution

This work is the first to employ VR technology to measure the visual exploratory components necessary to improve read-the-game ability in soccer. Our simple approach, which uses head rotational information obtained from the HMD to measure the VEA, can identify the head turn excursion range during real match simulations. Additionally, we offer insight into the relationships between the controller type, sense of presence, and impact on VEA performance. Concretely, for the consecution of a VR experience with a high sense of agency, utilizing full body kinesic natural mapping controllers to execute soccer actions inside VE provides a higher immersive effect compared with traditional input devices. Moreover, commercially available hardware can produce replicable results in an accessible programming environment. These design choices were selected for consecution of a prototyping testbed in future VR-based VEA training solutions.

### Related work

#### Read-the-game

Soccer players must discriminate surrounding actions in real-time based mainly on visual information. To make the best passing decisions, they must effectively explore the most extensive pitch area within a limited time span. Maximizing in-game passing decisions is achieved by correctly anticipating both teammates and rivals’ positions on the field. In this case, moving only the eyes is insufficient. However, moving the head and body can maximize the area covered in VEA. These elements are critical components of the read-the-game ability.

The term read-the-game has been referred to as “a player’s ability to anticipate future events from early components of an action sequence, which is an integral part of skilled soccer performance” [16]. A recent study on read-the-game and positioning on the pitch reduced unnecessary running and subsequent player fatigue [17]. In other words, a player must be able to quickly assess the situation to understand what is happening on the pitch and react effectively. This requires a certain level of perception, knowledge, and experience [18].

Related research suggests that in team sports, like soccer, players must: (a) resolve anticipation-coincidence motor problems (set of movements before the arrival of the ball and regulate these movements as the ball arrives), (b) make choices among information where potential solutions depend upon costs and benefits, and (c) manage varying courses and trajectories of players or the ball in conditions of decisional urgency [19]. Because these elements are essential for in-game decision-making and directly affect the outcome of team play, they are the backbone for collective games. Herein we evaluate VEA required to accomplish the best performance possible by focusing on elements (b) and (c) mentioned above.

Read-the-game is related to both vision and game intelligence [2]. The aim of this study is to elucidate new insights into the requirements for a technological solution that can measure VEA of the user in a comprehensive and meaningful way. Previously, a study administered a questionnaire to managers within English professional football to determine the qualities needed for specific positions. Managers noted that center back and center midfielders tend to have a better read-the-game ability [20]. Hence, we chose amateur players who played center back and midfield positions as the initial targets to develop and test our system.

Coaches intuitively understand the meaning of read-the-game. However, a method to measure, analyze, and train this ability in a quantifiable manner is lacking. This skill is often developed through repetitive practice, training drills simulating various game scenarios, and in-game experiences. In this study, we propose a new approach to evaluate and develop the read-the-game. ability.

#### VEA

Since we strive to define read-the-game elements as a subset of skills that can be measured and analyzed, we focus on identifying a quantifiable perceptual component of such a skill. Related research reported that “football [soccer] players adapt their movements to opportunities within the surrounding environment by engaging in VEA to pick-up information” [21]. Initially, this concept was defined by Jordet (2005) as “a body and/or head movement in which the player’s face is actively and temporarily directed away from the ball, seemingly with the intention of looking for teammates, opponents or other environmental objects or events relevant to the carrying out of a subsequent action with the ball” [10].

Roca et al. (2011) examined visual search behavioral differences between experienced and less-experienced soccer players using an eye-movement registration system. “The skilled players employed a search strategy involving more fixations of shorter duration in a different sequential order and toward more disparate and informative locations in the display when compared with the less skilled counterparts” [22]. Recent research demonstrated the importance of checking one’s shoulder, analyzing both the head turn frequency and head turn excursion of soccer players by implementing Inertial Measurement Units (IMUs) to quantify the players head exploratory movements [4]. We adopt a similar approach in a VR simulated setting while creating highly immersive and uniform in-game situation for all the users. Thus, the degrees of rotation of the body and head as well as FoR covered while performing visual explorations are assessed.

To obtain sufficient visual information during a match, soccer players must visually explore the most extensive area possible to form a mental picture of their surroundings. Additionally, they must understand the positions of their rivals, teammates, and the ball.

Players in team ball sports cannot perceive all the necessary task-relevant information without engaging in an active looking behavior. That is, players must move their heads and bodies actively [10]. Additionally, players cannot grasp more visual information than what is available in their immediate range of vision, which is limited to 180 degrees with turning the head or body [23]. Hence, players must engage in VEA.

One study involving 118 English Premier League players showed that those who perform a more extensive VEA before receiving and passing the ball forward are more effective than those with a lesser VEA [5]. These previous studies indicate that training solutions that measure and encourage players to engage in VEA are beneficial. Another benefit is that our system does not require that players physically practice on a pitch.

#### VR as a medium for sports perceptual training

We evaluate different available technological solutions for perceptual training. Specifically, our prototyping considers conventional video and VR. Although VR typically evokes images of games, fun amusement park experiences (i.e., roller coasters or leisure-oriented simulators), or cutting-edge entertainment, VR has a long history of research and scientific applications in a variety of domains. One such field is sports training.

Previously, we used VR to analyze rugby players’ ability to detect deceptive movements in an attacking player’s approaching run as well as the anticipatory skills of handball players [24]. VR provides superior interactivity considering the aggregate amount of actions required to perform during sports play compared to conventional video. VR offers a promising solution to better analyze the perception–action loop of the athletes. A follow-up study investigated the uncoupled and coupled perceptive judgment tasks of handball goalkeepers in two conditions: video clips and VE. The VE condition produced superior results [25].

A different study examined the capacity of VR to assess the anticipatory skills of cricket players. They assumed that VR is better suited than conventional video playback, suggesting that abundant perception elements are not present in traditional video media [26].

Specifically, research has found complex connections between the human optical system’s perceived information and subsequent decision-making in sports [27]. Hence, visual information is a basic element of decision-making in a sports context. To realize a meaningful and useful experience, the elements of presence inside VR have been fully assessed. The main advantage of VR is that the multisensory stimuli can be delivered only through this type of media.

#### Immersive VR and presence

In psychology, a benefit of VR as a training tool for sport-specific skills is that it provides the sense of presence to the user. The capability to experience an event as if the individual is immersed in real life plays a significant role in learning and transferring skills [28]. Hence, immersion and presence are vital for a VR system, especially for training purposes.

There is some disagreement on the precise definition on immersion and presence. However, sensorimotor immersion (hereafter simply immersion) refers to physical stimuli influencing the sensorial system and the sensitivity of the system itself to user inputs. “The level of immersion is determined by the number and range of sensory and motor channels connected to the virtual environment, and the extent and fidelity of sensory stimulation and responsiveness to motor inputs (for example, head and body movement, and hand gestures to make commands)” [29].

The psychological effect of immersion on the individual is the sense of presence, which can be defined as “the perceptual illusion of no mediation” [30]. Another widely accepted definition of presence is “the sense of being in a virtual space that is presented by technological means” [31] [32] [33]. That is, the individual’s subjective and personal sensation of being “there” exist in a virtually recreated world.

#### Measuring presence

Considering that a high sense of presence may be linked with skill training efficacy [28], we measured and analyzed (1) the degree that our system provides the sensation of being in VE and (2) how our system affects VEA and read-the-game skill training.

Assuming that the level of presence is individualistic and subjective, the most common and straightforward method to measure the sense of presence offered by a VR system is a post-test questionnaire. Numerous questionnaires have been developed. However, these vary widely in scope and appearance [34].

The most commonly adopted questionnaire was proposed by Slater, Usoh, and Steed (SUS) [35]. They suggested that both external and internal factors contribute to presence. External factors, which were identified based on existing research, were quality and resolution of displays, consistency of environment, interactivity, realistic self-representation, and simple connection between actors and effects [34]. The elements measured by the SUS questionnaire have been validated. They have been related to the relevant presence questionnaire (PQ). Both SUS and the PQ questionnaire measure the same compendium of presence-related elements [36].

A more recent questionnaire that included elements from other relevant presence questionnaires is the Igroup Presence Questionnaire (IPQ) [37]. After rigorous factor analysis on the initially proposed elements involving a large dataset with more than 500 participants in two waves, three subscales emerged as independent factors: Spatial Presence, Involvement, and Experienced Realism [31] [37]. This type of factor analysis is vital because the different elements that compose the presence construct can be identified, realizing a more detailed and factor-oriented evaluation of VR experiences. Currently, only the IPQ and the Witmer-Singer PQ questionnaires have been verified to be statically robust [38]. Additionally, the IPQ questionnaire has publicly validated translations, including English and Japanese versions. This study employs the IPQ due to its validation by the VR research community and availability in multiple languages. Because our test subjects were enrolled at the University of Tsukuba, we applied the Japanese version of the questionnaire. The specific items of each version of the questionnaire are available in S1 Appendix.

#### Repetition-based VR training and skill acquisition

We designed the VR implementation assuming that a technological solution can facilitate repetitive VEA training in a controlled environment to enhance skills such as read-the-game. In Sports Science, “a repetition is the lowest common denominator within the practice schedule, but is believed by many to have the greatest impact on skill acquisition” [39]. Even though training in a real-life scenario is ideal, VR can generate simulated in-game situations that are not feasible in real life [40]. For example, it is unrealistic to ask the whole crowd of a sold-out soccer match to be present at every practice, but this situation is useful to recreate a real soccer match scenario, where the support or antagonizing chants of the public may distract players. On the other hand, VR can infinitely recreate the away-game feel of a stadium filled with rival fans. Moreover, the probability of suffering an injury due to colliding with other players can be reduced or even eliminated in VR.

Continuous practice with a VR system may foster the read-the-game ability at an unconscious level. It has been suggested that when a player develops an ability, it tends to become automated as “players demonstrate little awareness of what underlies performance and typically are unable to give the reasons for their performances” [19]. Although measuring the long-term training effect is beyond the scope of this study, we set the theoretical basis that can be used in future studies.

#### Kinesics mapping and full body immersion

In the domain of HCI, when confronted with digitally mediated experiences such as video games or VR simulations, humans interact with the digital world via various types of controllers. Previous studies have proposed the concept of natural mapping, where the movement performed in a digitally mediated world is a faithful representation of real-world movement pattern. That is, the controller method is naturally mapped to the mental image of directional moves. Researchers have divided HCI input devices into four subtypes of controller methods: directional natural mapping, incomplete tangible natural mapping, kinetic natural mapping, and realistic tangible natural mapping [41].

Directional natural mapping is the simplest and most popular. In this modality, the user presses a directional button and the movement is performed accordingly. This type of movement is not natural to humans. However, it is nicely implemented and well understood given the wide adoption and longevity of this method.

In the case of specific motion controllers such as Sony PlayStation Move or Nintendo Wii, the type of natural mapping is described as incomplete tangible mapping as it is a partial/incomplete representation of the intended movement. The controller itself is an approximated physical representation of the virtually presented object, moving accordingly to the tracked motion of the controller device held by the user.

The third category is kinetic natural mapping. This style of controller offers a realistic representation of the user’s body movements by relying on motion sensing. An example is Microsoft Kinect, which is well studied in a variety of scientific applications in computer vision–based human motion tracking and recognition [42].

Tangible natural mapping offers a realistic experience where the user interacts with an analog representation of the interactive world such as a real ball in the case of a soccer game that the user kick or an actual trackable bat in a baseball game. Although this is often the preferred type of controller, it requires specific physical dimensions and space for the user to manipulate without risk. Because this study aims to realize a portable and low-cost VR trainer that can be deployed almost anywhere, natural mapping was not a viable option.

Herein we opted for the Microsoft Kinect controller since it offers an economic, reliable, and noninvasive solution to body tracking. Regarding the effect of the controller on the sense of presence inside VE, a previous study concluded that “the higher technological interactivity of motion-based systems (particularly Kinect) increases feelings of spatial presence, perceived reality, and enjoyment” [43]. Hence, the Kinect controller may realize a full body immersive VR experience with a superior sense of presence.

## Materials and methods

### Participants

To validate the technological proposal of this work, we conducted a voluntary experiment involving twenty-four (24) soccer players (7 females, 17 males, ages 19–30, mean age = 22.95, SD = 3.31). Twelve (12) of them were self-assessed beginners. They reported playing soccer as a pastime and had some experience during high school as a recreational activity. None reported more than two years of formal training. The other twelve (12) were self-assessed amateurs, actively playing in the university league and reporting between 7 to 15 years of formal training. All participants were students at the University of Tsukuba, Japan.

After consulting with the head coach of the university team, we chose players who were mainly defenders or midfielders. This decision was made considering the drill in the experiment, which simulated a typical in-game situation of passing to another player. Another factor was that a previous study showed similar opinions from professional English soccer managers [20]. To void the novelty factor, the experiment included beginning players instead of non-players as they had some experience playing the sport.

### Ethics statement

The experimental protocol was developed under the supervision of the University of Tsukuba, Center for Computational Sciences, with permission and evaluation of the Ethics Committee for Human Subject Research at the university.

All subjects signed a letter of consent of their own free will. They agreed to the research outline, method, necessity of research subjects, safety risks, and how to avoid potential harm through the protection of personal information, including recording of video. Additionally, they expressed their understanding that refusal to collaborate in this research would not be detrimental and that they could revoke consent to participant at any time without penalty. The test subjects confirmed that they could withdraw consent to provide data for a period of 365 days (1 year) or prior to publication in a journal without penalty.

The individuals whose image appears in this work provided written consent for the usage of personal information as per the Consent Form for Publication in a PLOS Journal.

#### Measuring VEA with HMD

Previous studies demonstrated that expert players tend to spend more time exploring more extensive areas of the field and less time fixated on the ball compared to less experienced players [44]. The VEA of players was measured while wearing a nine-degrees-of-freedom IMU’s during pre-season matches [4]. This study measured the VEA behavior of players with different levels of expertise in the same experimental conditions. A player’s experience varies during live play. However, VE can control the variability. Because maintaining a real match feeling was paramount, we used a VR headset.

The VEA of the soccer players was divided into three types: a) sequential exploratory activity, b) long exploratory activity, and c) 180-degree exploratory activity. A sequential exploratory activity is a compounded continuous sequence of exploratory searches in which the player’s face clearly is directed towards several distinct areas of the field before redirecting towards the ball. A long exploratory activity is a search in which the player’s face clearly is directed away from the ball for a second or more before redirecting towards the ball. A 180-degree exploratory activity is when a player’s face is clearly directed in the opposite direction of the ball viewed through an axis from the ball straight through the player’s body [10].

This study strives to analyze and train the player’s VEA behavior by promoting their engagement in both long exploratory activity and 180-degree exploratory activity before passing the ball to a teammate. Similar to previous research, the objective is to observe the head turn excursion before passing the ball to a teammate [4]. Consequently, we used the data obtained from the tracker regarding the orientation of the HMD. FoR seemed like a natural fit based on a previous work [11].

By dividing FoR into representative zones of the exploratory activity and calculating the total percentages of frames spent in a particular zone during visual exploratory searches, where the player is looking before a pass can be determined. Hence, gaze direction and head rotation to engage VEA were measured using HMD with Oculus CV1 and 360-degree FoR.

#### Zonal division

As this is the first VR-based system to measure the VEA component quantitatively, the score criteria of the VEA behavior was initially established. FoR of the HMD was divided into three key zones to identify the percentage of the time engaged in VEA before making a passing decision under rival pressure. To simplify the interpretation of the obtained data, we used the following zones:

*Zone 1*. This zone is right in front of the user. Eye fixation on the ball is possible. It is the area between 45° and −45° (315°).

*Zone 2*. This zone is the area where the user starts to engage in VEA looking away from the ball by engaging in significant head rotation to explore the surroundings in search of good passing options. The play enters into a long exploratory activity. It is the area between 45° and 90° to the right, and −45 (315°) and −90 (270°) to the left of the user.

*Zone 3*. This zone represents the 180-degree exploratory activity. The area is located at >90° and <270° clockwise. Because it includes the whole area behind the user, it requires full rotation of the torso and body to visually explore the surroundings.

**Fig 1:**
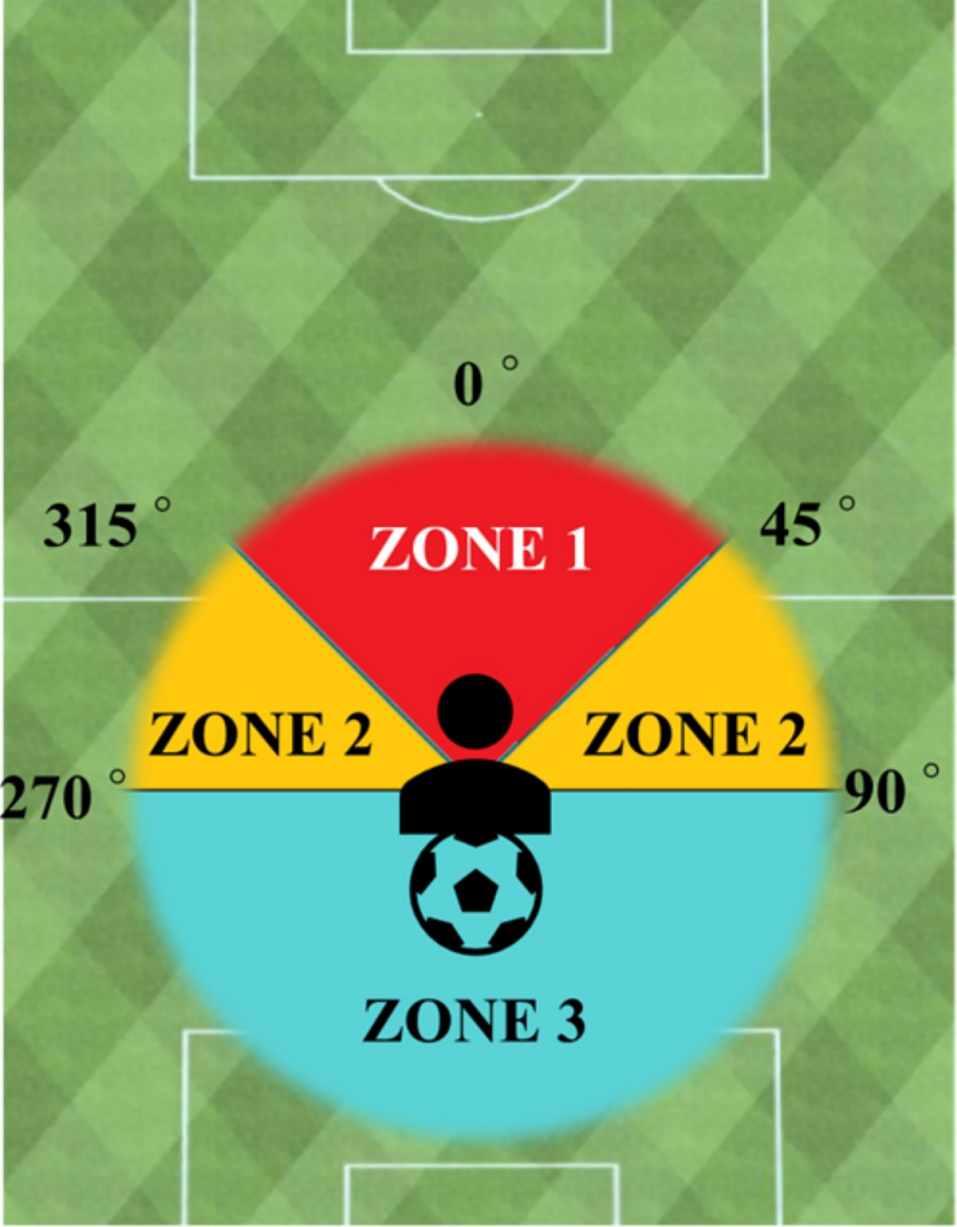
Zonal division of the visual exploratory activity.

The cumulative total percentage of the time spent looking at each respective zone can accurately measure the degree and type of VEA performed by a player (i.e., time engaged in significant VEA) during a simulator session. For this information, head directional data was obtained. Fig 1 shows a diagram of the zonal divisions.

#### Obtaining head rotational data from the HMD

For the visual and auditory feedback, we chose the Oculus Rift Consumer Version 1 (CV1). We measured the VEA by dividing the HMD FoR into three referential zones. The tracking system of Oculus Rift CV1 determined the gazing direction. The native tracking system of Oculus Rift CV1 extracted information about the head orientation of the player.

By extracting the Euler angles of the head orientation, the gaze direction was analyzed and compared. According to Euler’s rotation theorem, any 3D orientation can be produced by a single rotation about one axis through the origin. This axis–angle representation maps naturally to the space of unit quaternions as

$$q(v,\theta )=\left(\cos\left(\frac{\theta }{2}\right),v_{x}\;\sin\left(\frac{\theta }{2}\right),v_{y}\;\sin\left(\frac{\theta }{2}\right),\;v_{z}\;\sin\left(\frac{\theta }{2}\right)\right)$$ (1)

where *q(v*, *θ)* denotes a unit-length quaternion that corresponds to the rotation in *θ* radians about a unit-length axis vector *v =* (*v*_*x*_, *v*_*y*_, *v*_*z*_) [45]. Based on this, we extracted yaw, pitch, and roll coordinates from the center eye of the HMD to evaluate the direction of the user’s head frame-by-frame during the simulation.

In Oculus Rift CV1, a positive rotation is counterclockwise when looking in the negative direction of each axis. Pitch is the rotation around the x-axis, which is positive when pitching up. Yaw is the rotation around the y-axis, which is positive when turning left. Roll is the rotation around the z-axis, which is positive when tilting to the left in the xy-plane [46]. By extracting the quaternion angles of the yaw (y), pitch (x), and roll (z), and transforming into Euler angles, the orientation of the HMD’s center eye was obtained.

**Fig 2:**
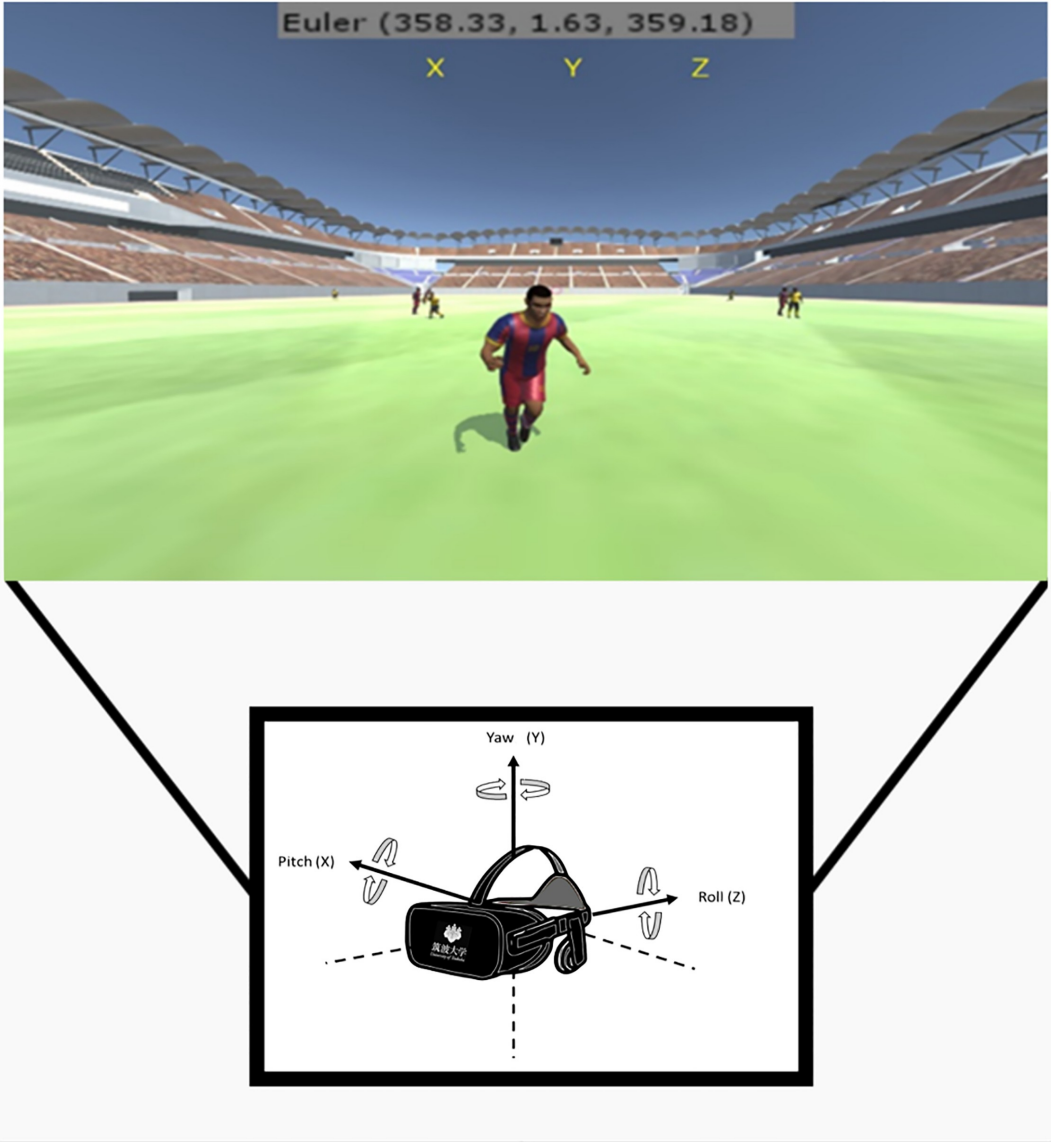
Yaw, pitch, roll, and Euler angles obtained from HMD.

After logging the Euler angles frame-by-frame and saving as a CSV file, the gaze direction was associated with the VEA zonal division whenever the user looks at each of the three corresponding regions. Fig 2 shows an image depicting the view of the subject with the related Euler angles. It should be noted that the Euler angles shown the top of the image were not displayed during the test. They were for development purposes only.

### Apparatus

#### Hardware

To realize a soccer training experience, we employed a combination of consumer- level devices. The chosen HMD is lighter than other mainstream HMD options. For example, Oculus Rift CV1 weighs 470 grams, whereas HTC VIVE, which is another candidate, weighs 555 grams [47]. The weight is a significant factor as our system requires players to rotate their heads repetitively and abruptly on some occasions similar to the motions in a real soccer match. Therefore, a heavier, bulkier HMD adds unwanted weight, making the experience less realistic and exerting an unnecessary physical load on the user. In addition, Oculus Rift CV1 has one tracker and fewer cables, whereas HTC VIVE requires tracking lighthouses to provide room scale to the user, negatively affecting portability and ease of use.

For the passing gesture, the user’s body motion was captured while performing a passing gesture with the leg using a Microsoft Kinect motion-sensing controller. There is the ample research supporting its use for human motion tracking and recognition [42].

Both devices (HMD and Kinect) were connected to a laptop PC, powered by one Nvidia GTX 980 GPU, an Intel Core I7 6700K CPU, and 16 GB of RAM, which processed the data from both the HMD and Kinect.

#### Kinetic body tracking and passing gesture recognition

To realize a full body immersive VR experience, a gesture recognition system was integrated with the kinetic body tracking. We used a Microsoft Kinect for Windows v2 motion controller.

Since the experiment involved passing the ball to a teammate, we built a training video database to recognize passing gestures and subsequent execution of a pass inside VE. The visual gesture builder (VGB) software was used to generate the data that our system used to detect the passing gesture at system runtime [48]. AdaBoost, an adaptive boosting machine learning algorithm, was used [49]. AdaBoost works with a Boolean value. That is, the algorithm determines if a passing gesture is performed and then triggers the corresponding action inside the simulation in the case of detecting the gesture.

The workflow to train the gesture detection algorithm required that a solution was initially created inside VGB using video clips of individuals performing the passing gestures captured with the Kinect and the Kinect Studio application. Eight collaborating individuals were recorded performing passing gestures to their front, back, both sides, and a random direction. To enhance the robustness of the training data, gestures were captured from random angles and locations. These clips were then imported to VGB.

The frames of the video clips in which the collaborating individuals performed passing gestures were manually tagged so that the machine learning algorithm detected when a passing gesture was executed. Then the passing gesture database was trained using the labeled clips, employing the AdaBoost algorithm, and testing it against similar clips of individuals performing random movements mixed with passing gestures.

**Fig 3:**
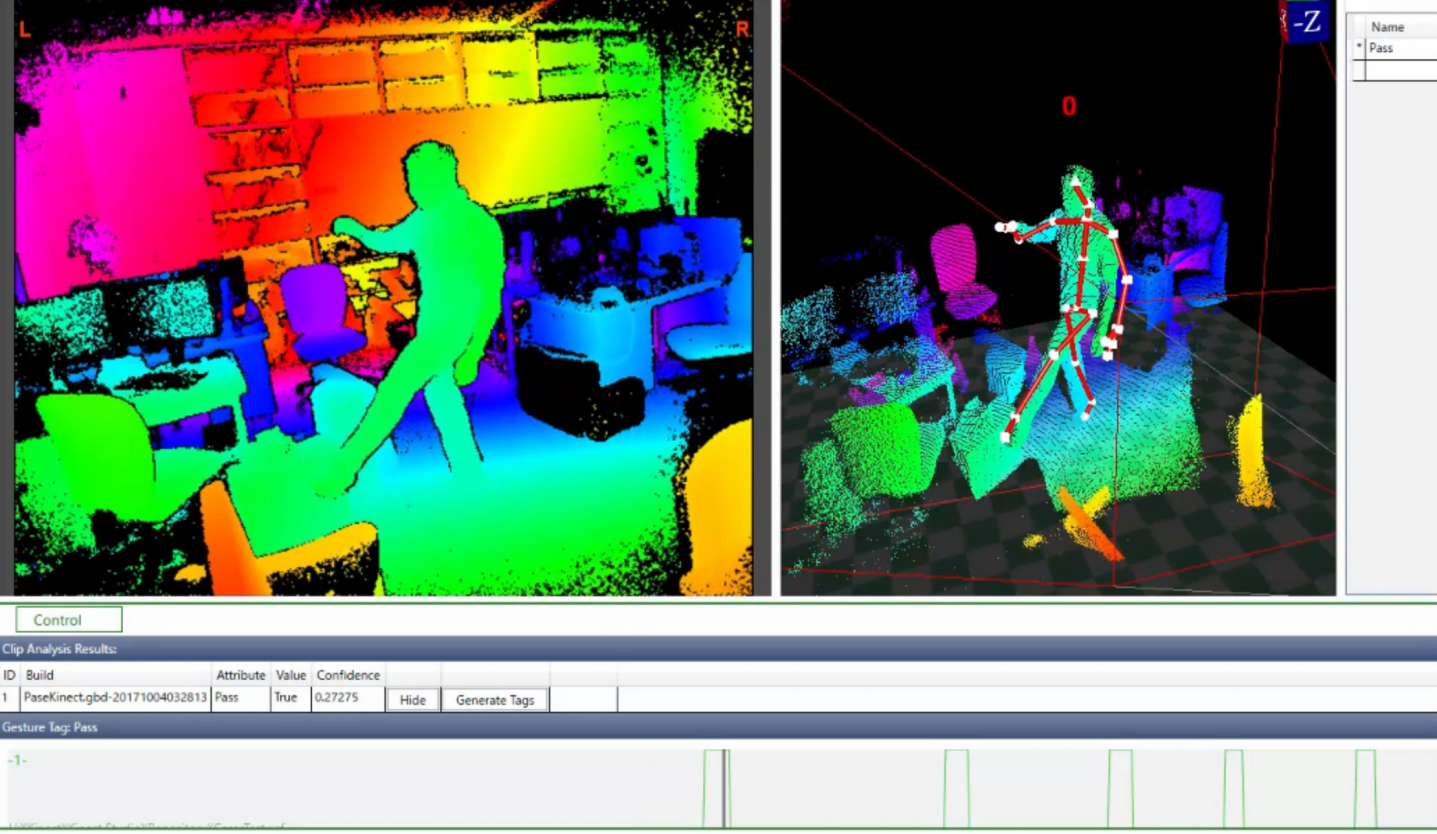
One of the eight video clips used to train the gesture recognition algorithm. The small blue lines in the timeline at the bottom of the image denote the manually tagged passing actions.

After training the data and carefully tagging the training clips, neither false positives nor failed detections occurred, indicating that our system correctly identified passing gestures. Fig 3 shows a screenshot of a video used for the training data where the passing gesture is tagged inside VGB.

**Fig 4:**
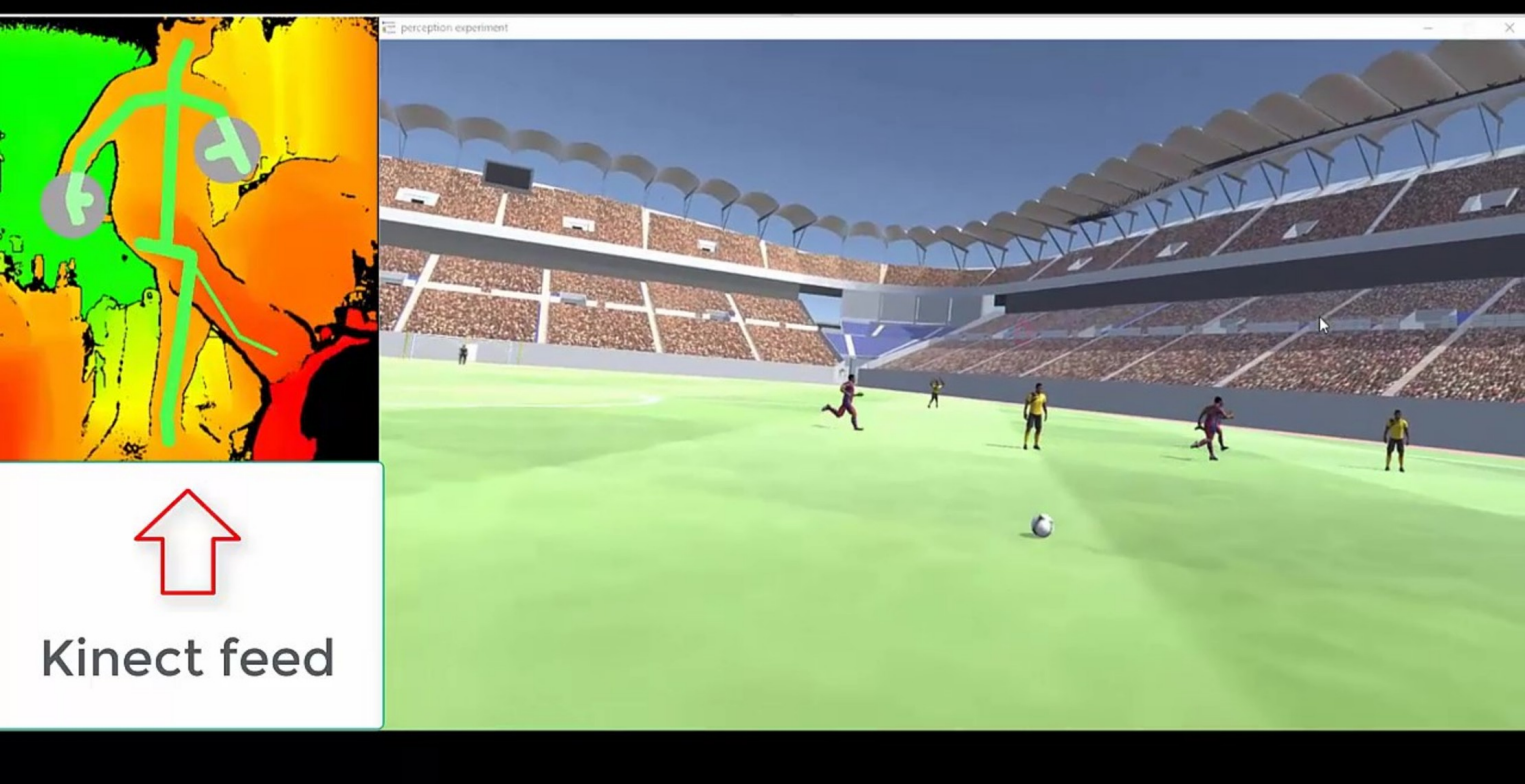
Kinect feed of the user inside the simulation. The left shows the image detected by the sensor of a user performing the passing gesture, while the right is the resulting ball pass inside VE.

The built passing gesture database was added to the VR simulation using the Unity Pro package for Kinect (Microsoft). The pass inside VE was performed every time the sensor detected a passing gesture and returned a positive Boolean value. Fig 4 shows an example of a pass in VE.

#### VE

Unity 3D, a widely adopted game engine, was used as the computer graphics engine. It has many assets. Because VE aimed to be a faithful representation of real soccer match situations, VE is a crowded soccer stadium to provide a strong sense of presence and exert task-related stress to the user.

#### Scale and dimension inside VE

One crucial aspect was the correct scaling of VE to convey a sense of being inside a real game because this affects the psychologically perceived sense of presence. Hence, the actual dimensions of the objects represented inside VE were used for the virtual assets.

**Fig 5:**
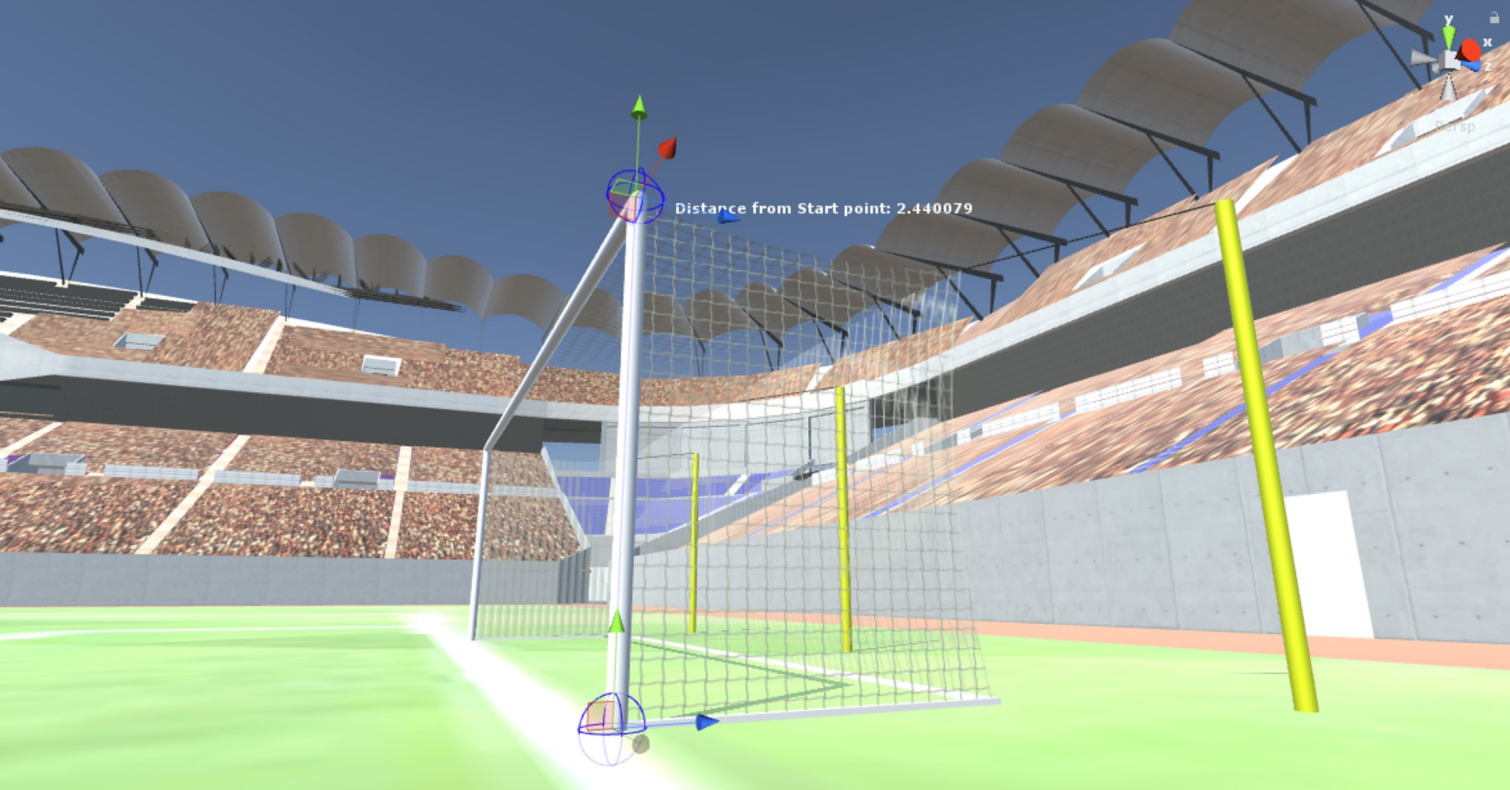
Goal post height measured with unity units, which are converted using a meter conversion tool. The real standard size is 2.44 m as established by FIFA.

Unity 3D was conceived such that one meter in real life was equivalent to one unity unit, which was the default measurement unit denomination employed inside the development environment. If the real measures of a given element were available, then such information was translated inside Unity while maintaining the required scale fidelity (Fig 5). Consequently, the user could see an accurate representation of real-life elements inside VE without hindering the sense of immersion. Specifically, we employed FIFA standard measures of the pitch to achieve the desired sense of scale [50].

**Fig 6:**
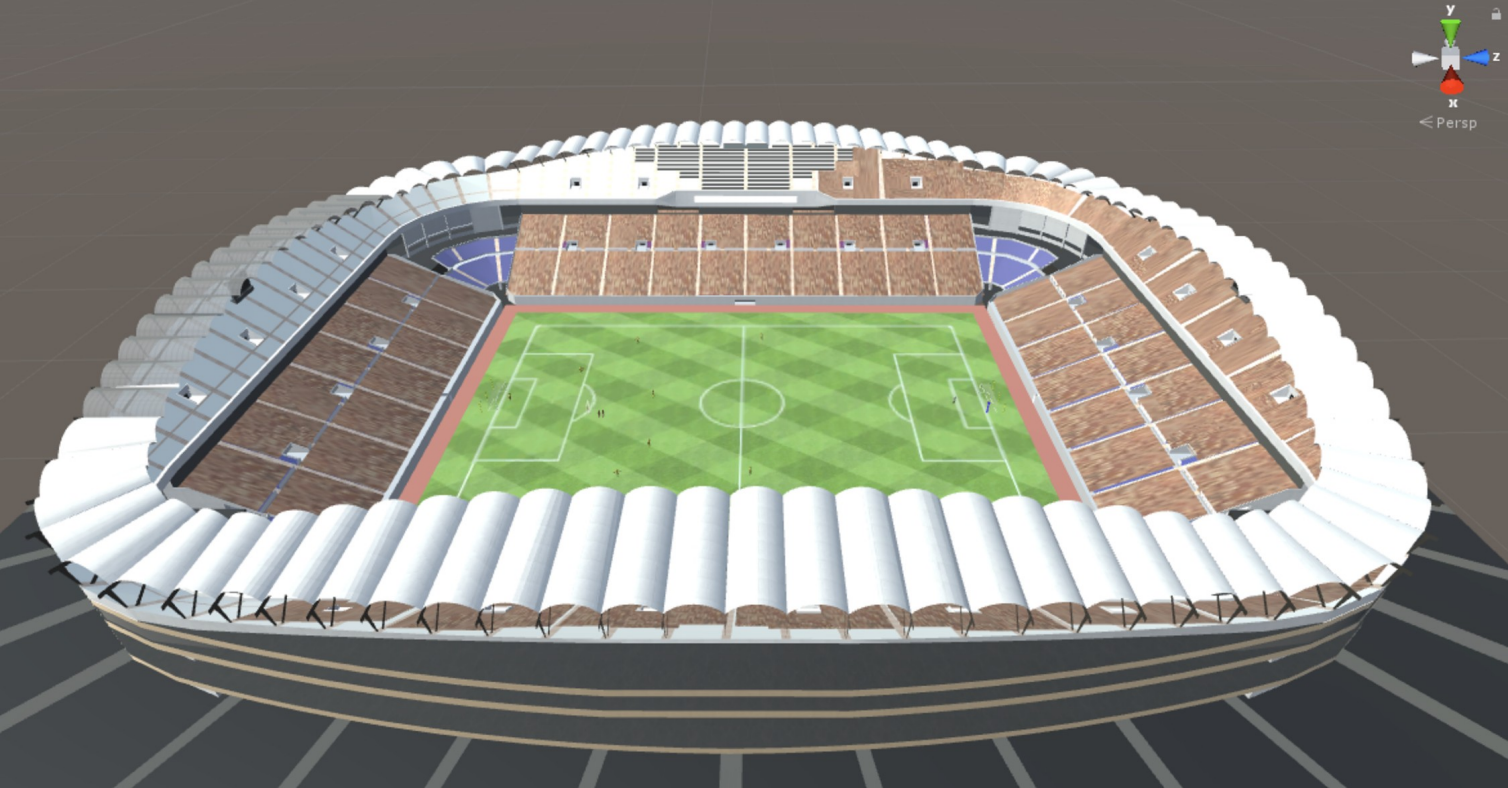
Kashima stadium model in unity editor.

Other elements, such as player model height were also considered. Furthermore, a one-to-one scale model representation from Kashima Stadium was used as the venue for VE. The SketchUp model (.skp) file was imported and fitted to scale with Unity’s coordinate system from 3D Warehouse [51]. Thus, the corresponding real-life dimensions of the venue could be measured, adjusted, and set (Fig 6).

#### Sound

The psychological effect of supporting fan crowds has been studied. In elite sports, screams and loud, effusive chants create an entirely different atmosphere on the pitch, especially in a decisive match. By contrast, an empty stadium during routine training sessions lacks distractions from passionate spectators. A previous study suggested that “although supportive audiences can inspire performers to excel when motivation would otherwise be lacking, audiences may also lead performers towards maladaptive self-monitoring and over-cautiousness when the stakes are highest” [52]. Hence, sound was a relevant element for the VE design. We chose the supporters’ chant of the Kashima Antlers, a popular professional Japanese soccer team, because the experiment conducted in Japan. A familiar sound may provoke a stronger cognitive impact.

Comparing the degree of psychological effect with and without a crowd sound is beyond the scope of this study. In the future, we plan to explore this variable. To help users become accustomed to VE while facilitating the sound volume level adjustment before experiencing the main test scene, other generic sounds were also presented during the tutorial scenes and menu navigation.

#### VE Content and scene structure

To maintain the sense of presence of the user and to familiarize the user to VE and the interface mechanics during the experiment, the simulation contained a navigational menu and two tutorial scenes before the main test scene. The title and content of the navigational menu and scenes present in our VE were as follows.

**Fig 7:**
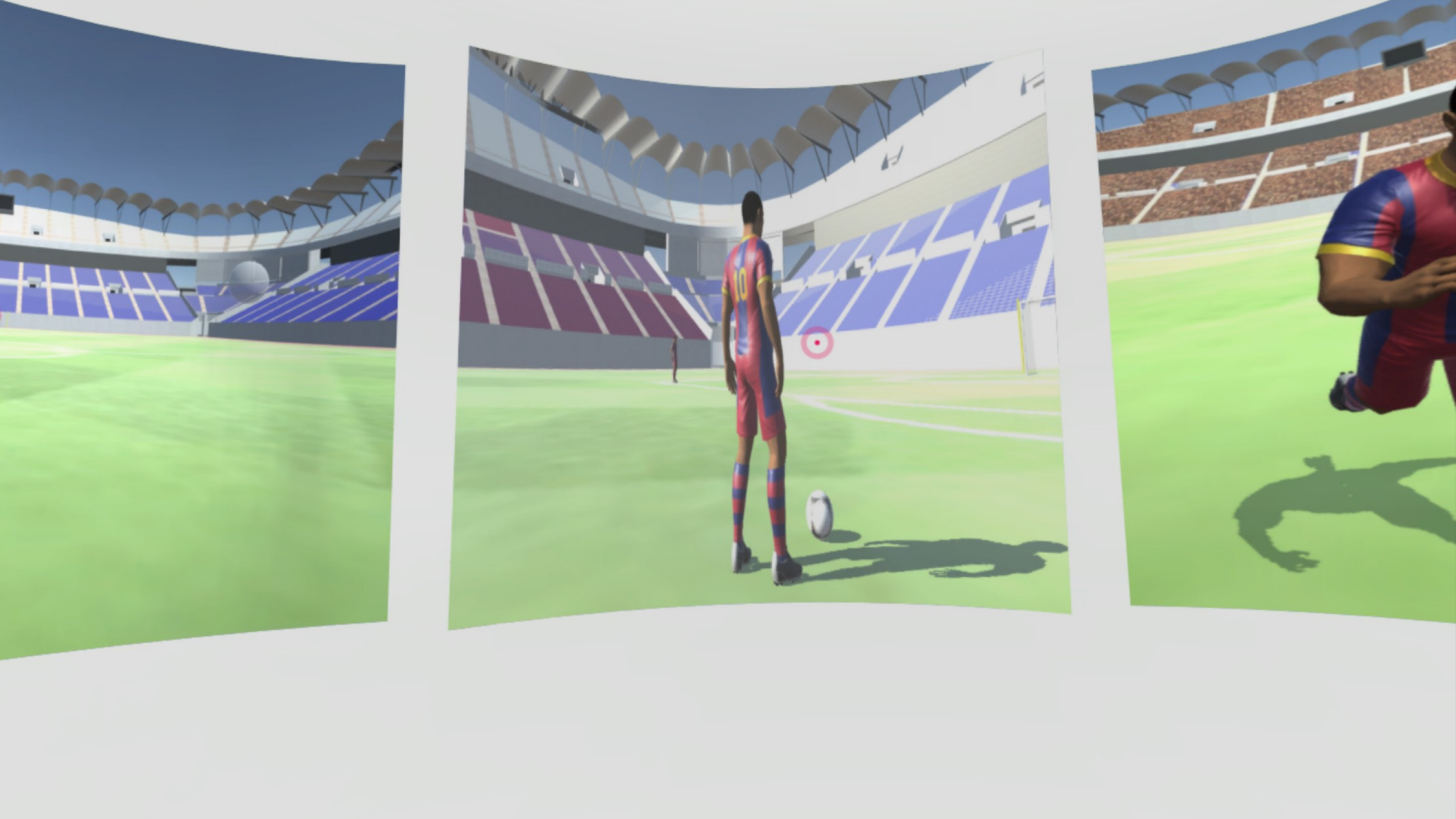
View of the Main Menu.

*Main Menu*. The user encounters a navigational menu from the moment he/she puts on HMD. It serves as a neutral ground for the user to become comfortable with VE and primary gaze and point mechanics. The center eye of HMD raycasts a small pink pointer, which acts as a visual reference to interact with virtual objects and identifies the center of their field of view (FOV). In front of the user, three scenes are displayed from left to right, which represents the order that they should access during the experiment. Fig 7 shows the user view of the Main Menu.

**Fig 8:**
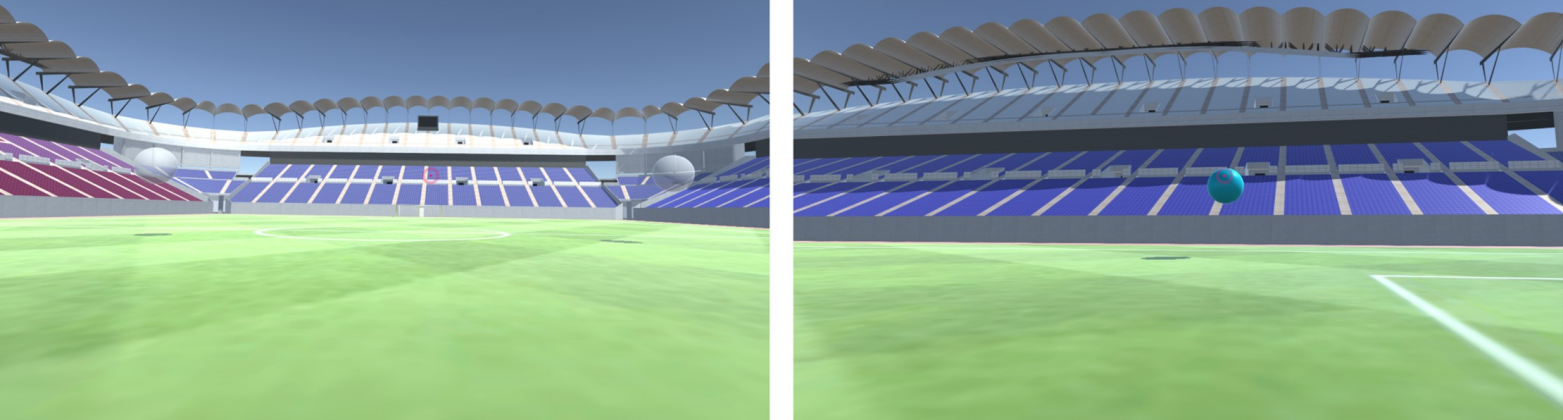
“Look at the spheres” scene. Left shows two white spheres in their initial state, while right shows the user gazing at a blue sphere.

Scene 1 is titled “Look at the Spheres” (Fig 8). It is intended to help the user acclimate to the gaze and point mechanics and become comfortable with the headset during active movement and overall head rotation dynamics. When the user enters this scene, he/she must search and gaze at four white spheres positioned in different locations to induce head and body rotation. The scene occurs in an empty soccer pitch with ambient sound of an empty soccer stadium.

Every time the user finds and points to the sphere with the HMD’s center eye’s visual pointer, the sphere changes to blue and a beeping sound is played through the headphones. Once the user successfully finds all four spheres, the scene returns automatically to the Main Menu and provides feedback about the sound volume.

**Fig 9:**
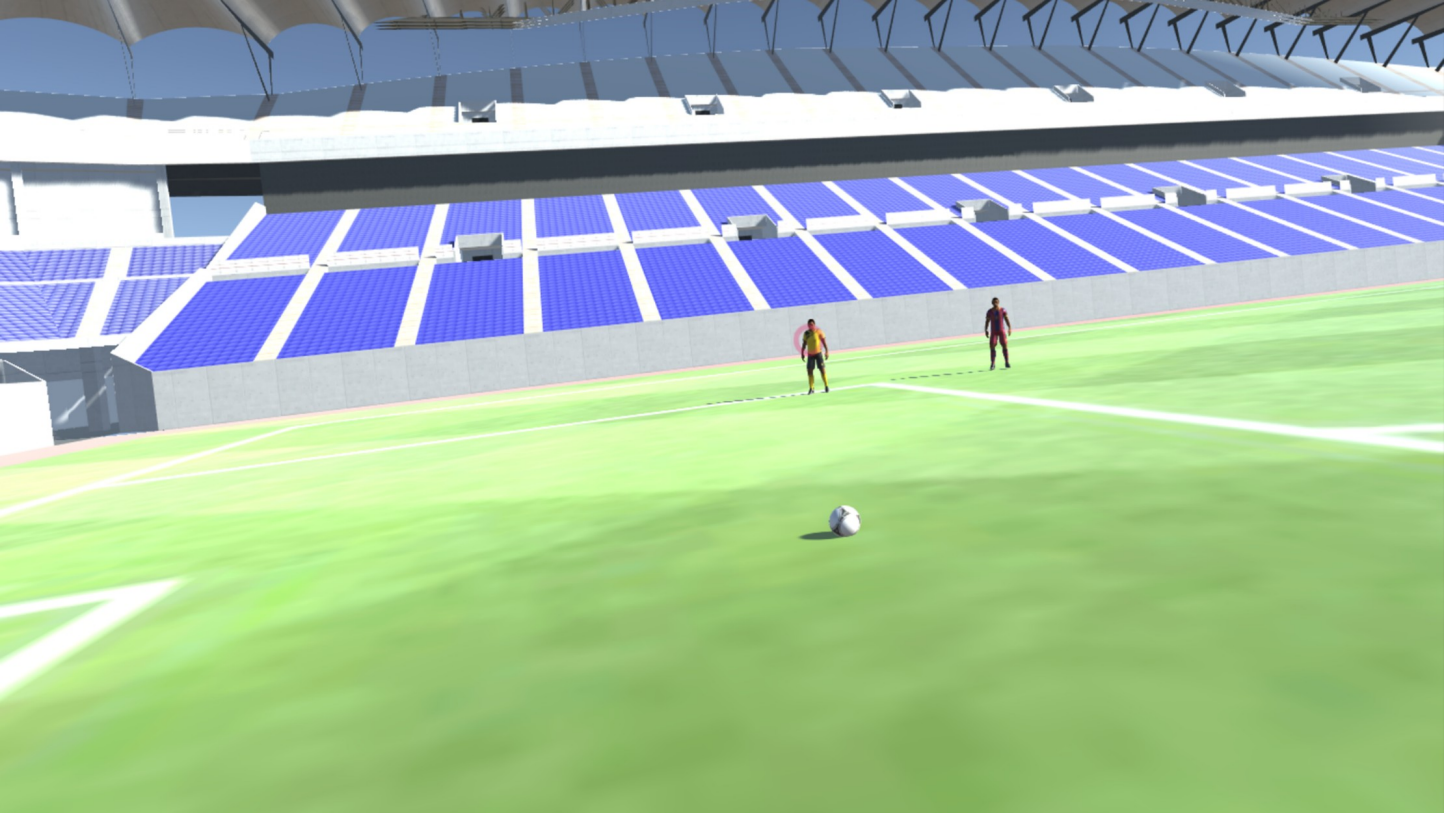
“Pass the ball” scene.

Scene 2 is titled “Pass the Ball” (Fig 9). In this scene, the user is in the same empty soccer pitch as in scene 1. The user faces three teammates dressed in yellow and three rivals in red uniforms. They are collocated in pairs of a teammate beside an opponent in three distinct locations. The objective is to familiarize the user to the players CG models used during the Test Scene. To learn the passing mechanics of the system, the user is asked to pass the ball successfully to the three teammates in yellow. Similar to real life, the user performs a passing gesture with the body. Every time he performs a passing gesture, a ball pass occurs towards the direction that the user is facing. If the pass is performed correctly and the CG model of the ball reaches a teammate, the teammate’s CG model disappears. Then the user must pass to another companion. If unsuccessful and the ball does not reach a teammate, the ball reappears at the user’s feet. The user performs this routine until the ball is successfully passed to the three teammate’s CG models. Then the scene jumps back automatically to the Main Menu.

**Fig 10:**
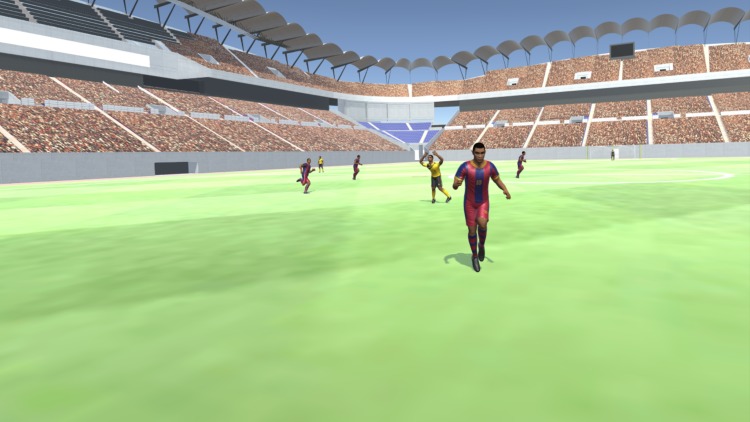
View of the “test” scene.

Scene 3 is entitled “Test” scene (Fig 10). As the name suggests, the VEA behavior of the user is logged and saved frame-by-frame. The user finds him/herself in the same position in the soccer pitch as in scene 1, but the stadium is packed with a cheering crowd of chanting Kashima Antlers fans. Now a real in-game situation unfolds in front of the eyes of the user. The user has possession of the ball and a rival player runs to try to steal the ball. The user must pass the ball to a companion in a favorable position. The user must engage in VEA to determine the best passing option quickly before the rival player steals the ball. When the user performs a passing gesture, a ball pass occurs in the same fashion as in scene 2. The passing decision is logged and saved in a CSV file for analysis, and the test scene ends.

#### Soccer in-game situation recreated in VR

For the simulation, a realistic soccer in-game situation must offer an accurate and meaningful experience to the player. Consequently, we recreated a set play provided by the Coach Manual published by Fédération Internationale de Football Association (FIFA) [53]. Because our objective was to incentivize the players to engage in VEA, we chose a set play drill used for combined technical/tactical training of zonal defense for defenders and midfielders. In this exercise, called “7 v 6 (8 v 6) game to work on regaining possession”, seven players defend high up the pitch to try and win the ball. The team of the user has six players plus a goalkeeper. Play always starts with the user in possession of the ball. The opposing team starts moving toward the user’s teammates to exert zonal pressure with man marking. The user is pressed by a rival player who comes running towards him/her to tackle and steal the ball. The user has less than seven seconds to make a passing decision before the rival reaches his/her position and steals the ball from his/her feet. Although seven seconds is an eternity in soccer, we wanted to provide ample time for the player to visually explore the surroundings in a context where they had no previous tactical information.

**Fig 11:**
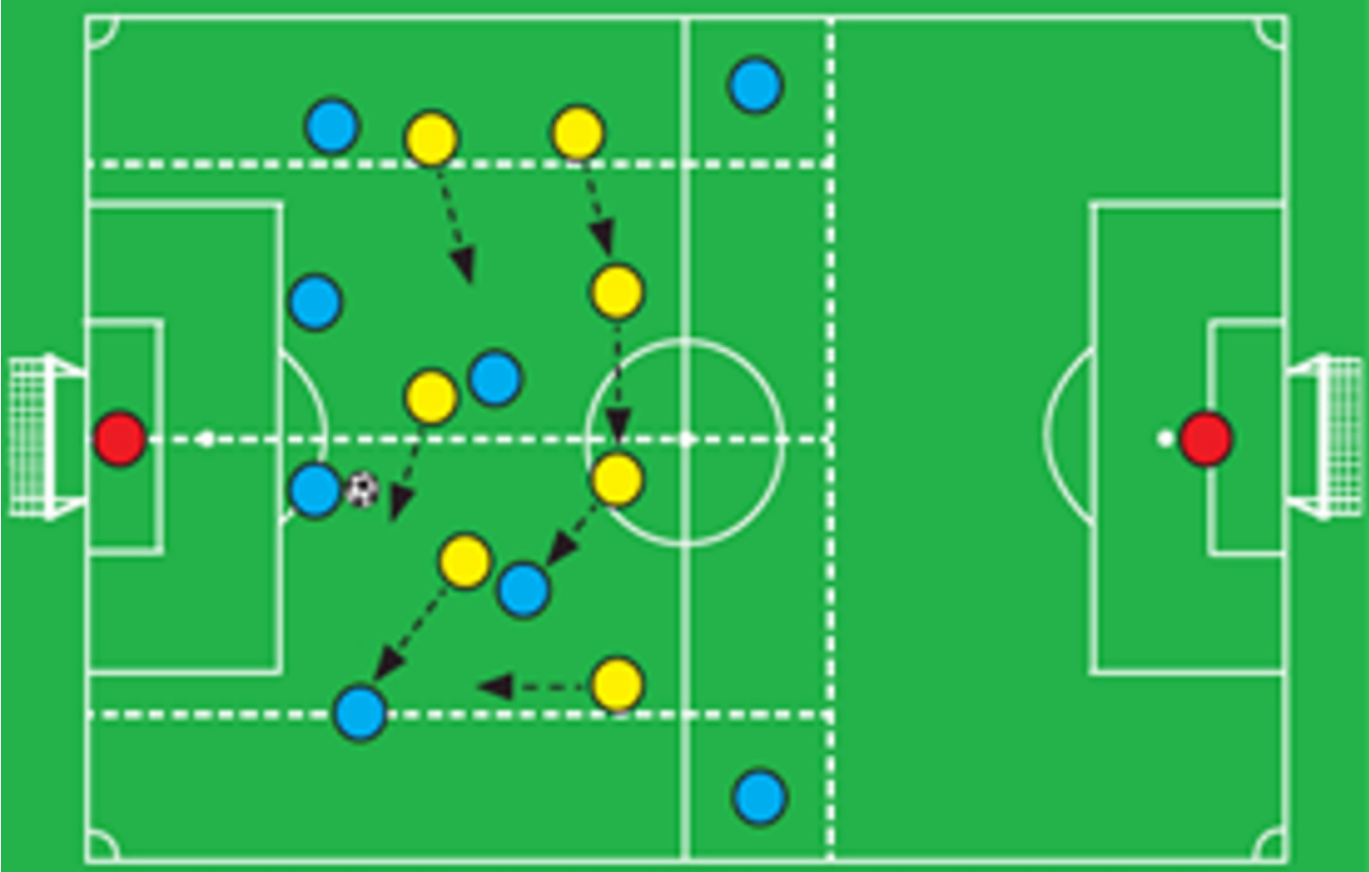
Variation of the set play drill in the FIFA coach manual [53] recreated in unity for our simulation. The user with the ball and his/her teammates are in blue, goalkeepers in red, rivals are in yellow. Arrows indicate the trajectories of the rivals during the scene.

Due to this high pressing scenario, the user must engage in VEA to gather as much visual information as possible about both rivals and teammates positions before making a passing decision. The only player without rival man marking and in a good place to receive a pass is located to the left of the user in zone 2, which is in the area between −45° (315°) and −90° (270°). To pass to the unmarked player, the user must engage in VEA in zone 2 to visually locate his/her teammate and make a passing decision (Fig 11).

### Experiment

Our experiment involved soccer players with different experience levels. The experiment aimed to validate the capacity of our VR system to measure the read-the-game ability of soccer players and the sense of presence provided to the user. Additionally, we evaluated two input devices (Kinect or Gamepad) to determine which one provides a more significant immersion experience.

### Procedure

The experiment lasted an average 20 minutes, with one trial per subject. Prior to signing a letter of consent to participate in the experiment, each participant was introduced to the system, read a detailed explanation about the experiment, and was given instructions. After providing consent, the user proceeded to the experimental environment.

In the experiment, the user stood 1.5 meters away from the Kinect sensor. He/she put on the HMD while the experimenter adjusted the straps until the HMD fit comfortably. When the VR simulation started, the participant was in the Main Menu scene. The participant was instructed to access the scenes in order from 1 to 3. The participants continuously gazed at the screen panel depicting the corresponding scene for 4 seconds in order to assess its content. Each scene was accessed from the Main Menu.

The tasks for scene 1 “Look at the Spheres” and scene 2 “Pass the Ball” were performed as explained in previous sections. Each time a task inside the scene was completed, the simulation returned to the Main Menu. The user could repeat the demo scene as many times as he/she wanted to become comfortable with the system mechanics. After completing the two tutorial scenes and reporting feeling confident with the system mechanics, the participant was instructed to prepare to access the scene 3 (“Test” scene), and to make the best passing decision using the same mechanics introduced during the two tutorial scenes. Once the participant accessed the scene, the gazing direction was logged and saved frame-by-frame for posterior analysis.

**Fig 12:**
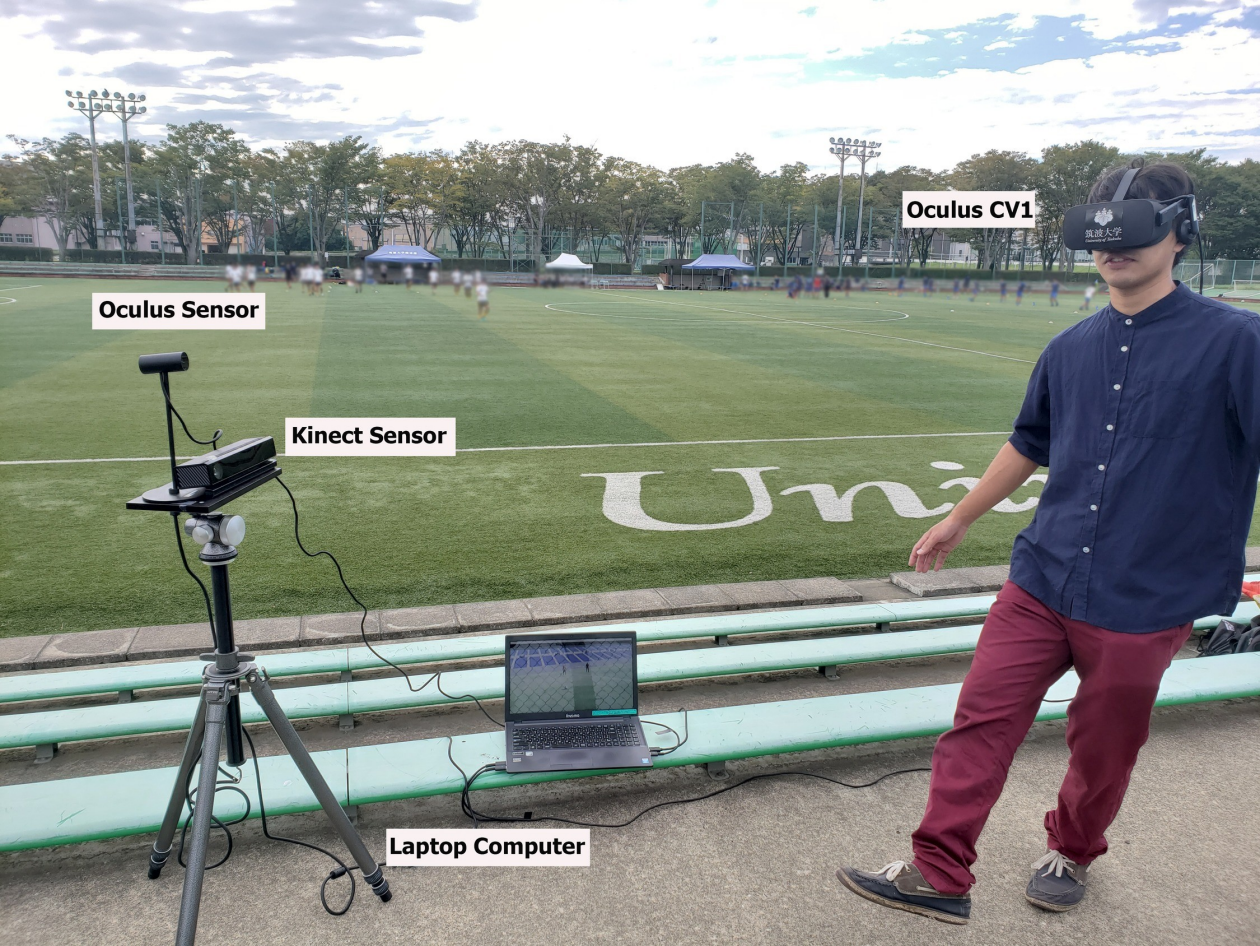
Volunteer experiments the VR simulation: Making a passing gesture inside VE. The image highlights the main components of the setup.

The simulation ended when the ball was passed. Then the experimenter helped the user take off HMD. Fig 12 shows a volunteer experiencing the simulation.

Once the system experiment ended, the user answered the IPQ questionnaire [37] in the mother tongue of the user. Specifically, participants completed the Japanese version of the questionnaire (S1 Appendix). To gather additional feedback about the user experience, there was an open comment section. The test subjects were also asked to describe their prior gaming experience and familiarity with HMD and VR systems.

## Results

### VEA score

The VEA score data of each subject was the measurable component related to read-the-game. Using the VEA zonal division, we focused on two patterns: long exploratory activity and 180-degree exploratory activity. The total percentage of the frames spent visually exploring zone 2 and zone 3 before making a pass represented the VEA score. Table 1 shows the VEA scores and skill level by participant.

**Table 1:** VEA scores by test subject during the “Test” scene, paired by their level of experience.

Test Subject	Level	Gender	VEA
Subject 1	Beginner	Female	22
Subject 2	Beginner	Male	20
Subject 3	Beginner	Male	3
Subject 4	Beginner	Male	48
Subject 5	Beginner	Male	46
Subject 6	Beginner	Female	53
Subject 7	Beginner	Male	37
Subject 8	Beginner	Male	49
Subject 9	Beginner	Female	0
Subject 10	Beginner	Female	25
Subject 11	Beginner	Female	39
Subject 12	Beginner	Male	28
Subject 13	Amateur	Male	58
Subject 14	Amateur	Male	41
Subject 15	Amateur	Male	29
Subject 16	Amateur	Female	50
Subject 17	Amateur	Male	50
Subject 18	Amateur	Female	51
Subject 19	Amateur	Male	74
Subject 20	Amateur	Male	73
Subject 21	Amateur	Male	25
Subject 22	Amateur	Male	15
Subject 23	Amateur	Male	54
Subject 24	Amateur	Male	78

Amateur players showed a higher average VEA score (mean score, 49.83; SD, 19.86) than beginner players (mean score, 30.83; SD, 17.61). To determine if the difference between groups was significant, we performed an independent samples t-test.

**Fig 13:**
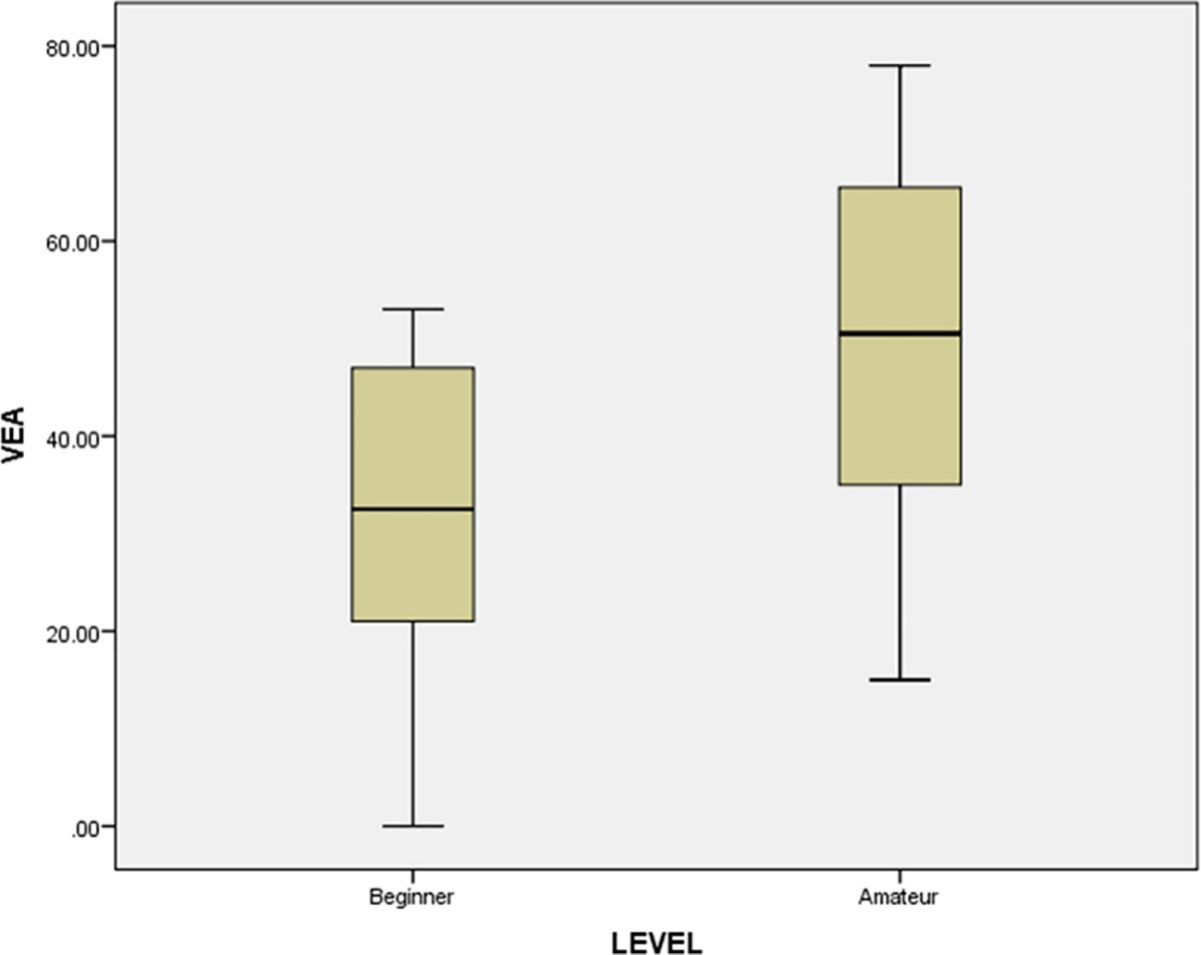
VEA mean score graph of beginner versus amateur.

To identify deviations in the variance by group, Levene’s test was used to evaluate the equality of variance (p-value = 0.931, 95% confidence). Since the p-value was significant (> 0.05), we assumed equal variances for the t-test. The obtained Sig. (two tailed) value for this sample was 0.021. The 95% confidence interval of the difference (−34.89628 to −3.10372) confirmed that the VEA scores by group were statistically significant (Fig 13).

### Sense of presence (IPQ)

The IPQ scores were analyzed as a composite measurement of presence based on 14 questions presented in a seven-point Likert scale format. Consistent with the literature [54], each item ranged from 0 to 6 and the total scores ranged from 0 to 84. Additionally, factor analysis performed by the iGroup (creators of the IPQ) defined three subscales: Spatial Presence, Involvement, and Experienced Realism [31] [37]. These were measured independently along with a fourth general question measuring the general sense of presence (GP) experienced by the user. Table 2 shows the results of the IPQ scores.

**Table 2:** IPQ scores.

Test Subject	GLOBAL	GP	SP	INV	REAL
**Subject 1**	**50.00**	**3.00**	**24.00**	**9.00**	**14.00**
**Subject 2**	**41.00**	**3.00**	**17.00**	**12.00**	**9.00**
**Subject 3**	**70.00**	**6.00**	**24.00**	**21.00**	**19.00**
**Subject 4**	**43.00**	**5.00**	**17.00**	**12.00**	**9.00**
**Subject 5**	**47.00**	**4.00**	**20.00**	**15.00**	**8.00**
**Subject 6**	**55.00**	**5.00**	**25.00**	**18.00**	**7.00**
**Subject 7**	**34.00**	**3.00**	**13.00**	**11.00**	**7.00**
**Subject 8**	**45.00**	**5.00**	**20.00**	**14.00**	**6.00**
**Subject 9**	**73.00**	**6.00**	**23.00**	**25.00**	**19.00**
**Subject 10**	**53.00**	**5.00**	**23.00**	**15.00**	**10.00**
**Subject 11**	**59.00**	**6.00**	**25.00**	**16.00**	**12.00**
**Subject 12**	**61.00**	**5.00**	**22.00**	**18.00**	**16.00**
**Subject 13**	**49.00**	**5.00**	**18.00**	**14.00**	**12.00**
**Subject 14**	**44.00**	**4.00**	**20.00**	**12.00**	**8.00**
**Subject 15**	**53.00**	**5.00**	**20.00**	**17.00**	**11.00**
**Subject 16**	**68.00**	**6.00**	**24.00**	**21.00**	**17.00**
**Subject 17**	**47.00**	**2.00**	**23.00**	**13.00**	**9.00**
**Subject 18**	**51.00**	**5.00**	**21.00**	**11.00**	**14.00**
**Subject 19**	**51.00**	**4.00**	**19.00**	**16.00**	**12.00**
**Subject 20**	**41.00**	**4.00**	**16.00**	**11.00**	**10.00**
**Subject 21**	**67.00**	**6.00**	**29.00**	**18.00**	**14.00**
**Subject 22**	**68.00**	**5.00**	**25.00**	**22.00**	**16.00**
**Subject 23**	**43.00**	**5.00**	**17.00**	**10.00**	**11.00**
**Subject 24**	**48.00**	**5.00**	**18.00**	**17.00**	**8.00**

GLOBAL: global score of the composite measure of the IPQ questionnaire, GP: general presence, SP: spatial presence, INV: involvement, REAL: sense of realism.

The average value of the composite measure of the IPQ was high (GLOB mean, 52.5417; SD, 10.54; scale range, 0–84), suggesting that the users felt somewhat present inside VE.

Looking at the three subscales and the fourth isolated factor of GP, GP was high (GP mean, 4.6667; SD, 1.09014; scale range, 0–6). This pertained to the subjective sense of being there experienced by the test subject. It had high loadings on all three subscales, and had an especially strong loading on spatial presence (SP) [31] [37].

SP also had a high average value (SP mean, 20.9583; SD, 3.70052; scale range, 0–30). This referred to the sense of being physically present in VE conveyed by the VR system instead of just acting as an external entity in a relationship with VE [15].

Regarding Involvement (INV), the mean value was slightly high (INV mean, 15.3333; SD, 4.15636; scale range, 0–24). This measured the amount of attention given to VE by the user and how involved he/she was with the VR experience instead of feeling distracted by the real world around them [15]. This measurement is of interest when selecting the most adequate type of controller for the system.

For realness (REAL), the average value was neutral (REAL mean, 11.5833; SD, 3.80979; scale range, 0–24). This measurement was related to the experienced degree of subjective realism inside VE [15].

There was no statistical difference between the IPQ scores when comparing beginners with amateur players.

### VEA and presence correlation in VR

One of the main reasons for selecting VR is that it may evoke real-like soccer match experiences with the subsequent cognitive stressing elements that only a competitive match can provide. However, if the user cannot interact with the environment similar to that in real life, the performance will deviate from a real scenario. In short, VR training can only be meaningful if sensory stimuli are sufficiently realistic.

To compare the effect of the input option in our system and the impact on the performance of soccer players, we employed an approach to link the sense of presence provided by the system with the VEA performance of the test subjects. We first performed a correlation test between the VEA scores and the IPQ results of each subject.

We analyzed the data in the scenario where the controller was used as an input option to trigger the pass inside VE. Before making a correlation test, the distribution of the data was evaluated. We confirmed normality both by visual observation of the histogram and a Shapiro-Wilk test applied to VEA and composite IPQ values (total sample, VEA p = 0.667 and GLOBAL IPQ p = 0.159).

We applied a correlation test to see which presence components were related with the VEA performance of the users. A strong significant correlation was observed between the composite GLOBAL IPQ test score and the VEA score (r = −0.542). The subscales correlations were also statistically significant, except for the subjective sense of general presence. We used the criteria established by Cohen (1988) to determine the intensity of the relationship due to the correlation test [55] (Table 3).

**Table 3:** Pearson correlation test (with Kinect).

VEA Score Correlation	GLOBAL	GP	SP	INV	REAL
**Pearson Correlation**	−0.542**	**−0.197**	−0.452*	−0.420*	−0.547**
**Sig. (2-tailed)**	**0.006**	**0.357**	**0.027**	**0.041**	**0.006**
**N**	**24**	**24**	**24**	**24**	**24**

** Correlation is significant at the 0.01 level (two-tailed)

* Correlation is significant at the 0.05 level (two-tailed)

Next we compared the correlation values using two different inputs (Kinect vs. Gamepad). The experimental data for the Gamepad was obtained from our previous study [11]. This study used the same system and methodology introduced in the referenced paper except for the input device.

In this variant of the system, the user performed the passing action by pressing a button of the Gamepad controller instead of performing the passing gesture with his/her body. The previous study was done with ten beginner and amateur self-assessed soccer players. Both studies used the same protocol.

In this study, the VEA and IPQ scores were not correlated significantly. Additionally, the performance and three of the four factors of the presence composite were not correlated. Interestingly, only a strong negative significant correlation r = −0.668 was found between the factor of Involvement and VEA performance [11]. We hypothesized that the lack of a strong relationship between VEA and general IPQ metrics might be due to the user’s feeling of playing a video game instead of living in a real-like soccer match experience. The Gamepad forced the player to execute actions unnatural to soccer like pressing a button, reducing the sense of presence compared with gesture-based inputs (Table 4).

**Table 4:** Pearson correlation test (with Gamepad).

VEA Score Correlation	GLOBAL	GP	SP	INV	REAL
**Pearson Correlation**	**−0.481**	**−0.106**	**−0.137**	−0.668*	**−0.434**
**Sig. (2-tailed)**	**0.159**	**0.771**	**0.706**	**0.035**	**0.210**
**N**	**10**	**10**	**10**	**10**	**10**

* Correlation is significant at the 0.05 level (two-tailed)

## Discussion

The experiment reveals a difference in the VEA behavior by skill level. Specifically, more experienced players show a higher VEA. Experienced players tend to cover a wider area as they rotate their heads and body before making a passing decision. These results are consistent with previous findings in which young inexperienced players show a lower visual exploration frequency than more experienced passing experts [56].

However, the distractor effects derived from the technological novelty felt by some test subjects may be a factor. For some subjects, this study was their first encounter with VR technology. The three test subjects with the highest VEA score in the beginner category had the highest level of gaming experience and were self-described as avid gamers (subjects 4, 6, and 8). This gaming background may compensate for the lack of soccer-related experience. The influence of the novelty effect should be further investigated.

Previous research showed the importance of understanding the total radial distance covered by a player’s head during a head turn [57][58]. Additionally, the various benefits of visually scanning the surroundings before receiving the ball have been reported [9][23][5]. An alternative technological approach employed a laptop computer and four monitors placed in FoR of the player. These displayed possible passing options. However, this setup gives the test subject a predefined idea of where to look for relevant information, conditioning his movement to predefined vectors [58]. By contrast, for the VR simulation in this paper, the whole FoR area was an option for the player. Hence, the 360-degree head excursion behavior could be tested before making a pass. It is important to highlight that the subject did not have prior knowledge of the placement of the virtual players before starting the VR session. This design decision forced the VR users to rely on visual information under strict spatiotemporal constraints. Nevertheless, this configuration eliminates other factors relevant to in-game decision-making such as prior tactical understanding of the teammates/rivals-expected behavior according to previous actions, which build up to the present game situation.

Due to the flexibility of VR as a medium allowing for quick changes to the simulated content, different in-game scenarios with longer play scenes are possible in the future. This would allow the player more time to adapt to the intrinsic game dynamics in a realistic manner. Moreover, exploring the variability of VEA behavior under different specific in-game situations and set plays that resemble competitive situations should be considered.

To confirm the system effectiveness as a training tool, it is also important to perform skill transfer tests to determine whether repetitive usage of the VR simulator leads to substantial improvements in the on-field performance. A good option is a variant of the methodology used by Pocock et al. (2017) to confirm the effectiveness of the PETTLEP imagery intervention in the improvement of VEA [21] while considering the specifics of the proposed system.

With regard to IPQ results, the system can provide a satisfactory sense of presence to the user. The somewhat lower sense of realness experienced (comparing IPQ to other subscales) may be because the CG world does not have the highest grade of photorealism. This may affect the cognitive impression of what is real. Hence, the images presented to the user inside VE are perceived as part of a synthesized world instead of a real one.

During our previous study using Gamepad as an input option, the only statistically significant relationship was a negative correlation with the sub-scale Involvement [11]. With Kinect, a negative correlation is observed in the IPQ composite results and three of its four subscales. The strong negative correlation decreases with VEA performance as the sense of presence is incremented.

Without a direct comparison between VR and the on-pitch VEA performance, which input option offers a VEA value that is closer to reality remains unclear. However, we hypothesize that the Kinect configuration is more accurate because an incremented interactivity may also convey a better realism of the stressors placed intentionally in VE (e.g., the cheering crowd, approach of a pressing rival, and the necessity for an effective biomechanical response). As suggested by Slater and Usoh (1994), habitation in a virtual world is recognized by our body becoming an object in the portrayed VE [59]. Concerning this assertion, our system provides a suitable setup for a highly immersive VR soccer experience, allowing the player’s virtual body to exist and feel as if the player is inside a real game with decision-making impairment due to a highly stressful competitive environment representation.

We also recommend testing the effect of multimodal stressors inside VE (e.g., with and without crowd sound). This may help investigate the relationship between stress and VEA performance.

The sample size for this experiment is somewhat limited. Despite being consistent with other studies on VEA, our results are mostly inferential with respect to the difference in means of the VEA score between self-assessed amateur and beginner soccer players. For a more representative result, the experiments need to be performed with more subjects and more observations in different in-game situations.

Additionally, the skill gap between the two groups is small. In the future, we would like to compare elite professional players and beginners. This should lead to a more significant analysis of the difference in experience related to VEA behavior.

The feedback of the players identified some challenges that should be addressed in the future. First, regarding the hardware and setup, three test subjects commented that they were somehow aware of the cable of Oculus CV1 during the VR simulation. One player said, “Sometimes, I was worried not to pull or become entangled with the cable while rotating my body.”

These comments suggest that a potentially negative impact of cognitive distractors on the sense of presence, especially in the sense of involvement. Since this subscale measures the amount of attention devoted to VE while unaware of the real-world element, it is highly possible that this type of distractors severs the concentration of the user, disconnecting his/her mental link from VE momentarily. Therefore, we would like to try untethered HMD solutions in the future to preserve the simplicity of the setup.

Another type of comment was related to the passing mechanics, specifically the passing vector direction estimation. Our system relies on gesture detection to initiate the ball passing action inside VE. However, the passing direction is determined based on the orientation of the center eye of HMD at the instant the input sensor registers a passing gesture. Considering the natural biomechanical motion of soccer players when passing, it is hard to maintain the head oriented towards the precise direction as intended. One user commented, “It was somewhat hard to make a precise pass because I couldn’t maintain my head correctly oriented towards the desired teammate.”

Even though most players adapted and performed well the passing action after a few tries during tutorial scene 2, other approaches to estimate the ball trajectory should be considered in the future.

## Conclusions

We introduce and test a novel VR system to measure and train the read-the-game ability of soccer players. By measuring the visual exploratory skills to make the most accurate in-game decisions, we propose an alternative technological approach in the sports science domain by analyzing the visual component of players’ read-the-game ability. We also consider both the psychological (i.e., sense of presence) and HCI (i.e., Gamepad versus Kinect) aspects in the design and implementation. The resulting impact on the VEA behavior depends on the input option.

### Read-the-game and VEA

Regarding the sports science aspect of our study, we tested and validated the capacity of our system to measure the visual component of read-the-game. The tested system measured the engaged VEA during a VR session by tracking the head rotation of the user frame-by-frame with HMD. Furthermore, during the system verification test, we observed a significant difference between amateur and beginner players. More experienced players more actively rotated their heads and bodies while performing visual explorations compared to less experienced ones.

### Presence

To realize an immersive VR system that may maximize the VEA training and analysis, we examined the level of immersion provided by the proposed method by focusing on its psychological byproduct “presence.” We measured the sense of presence elicited by our system through the IPQ Presence Questionnaire applied to beginner and amateur soccer players. Based on the questionnaire results, we can conclude that a full body VR simulation presents a promising option for immersive VEA training.

### Kinect and full body immersive VR

To further understand the effect of different input options in the sense of presence provided by a VR system in a soccer simulation context, we compared two types of input configurations. When the input device was a Kinect system, a strong negative correlation between full-body interaction and VEA performance was found. By contrast, such a correlation was nonexistent when using a Gamepad controller.

The consensus is that kinetic controllers are better for immersive VR than traditional button-based input devices. However, we were surprised to see such a statistically significant correlation between the type of controller and VEA performance. Based on this, the VR system presented in this study with the kinetic controller configuration is an adequate approach to measure VEA while evoking the tension and stress derived from feeling immersed in a competitive soccer match. Consequently, its prolonged use may allow soccer players and even coaches to analyze, train, and develop the VEA skill required to enhance the read-the-game ability. The added value that the system offers with respect to portability given the hardware provides flexibility in terms of when and where the VR training session can be performed. In other words, VEA training is possible anytime, anywhere.

## Supporting information

S1 Appendix Questionnaire. (DOCX)

S1 File (SAV)

S1 Movie (MP4)
