# Supplementary material for: Read-the-game: System for skill-based visual exploratory activity assessment with a full body virtual reality soccer simulation
Source: PLoS One. 2020 Mar 17;15(3):e0230042. doi: 10.1371/journal.pone.0230042 (PMC7077991; doi:10.1371/journal.pone.0230042)
Supplement: S1 Appendix — (DOCX) [file pone.0230042.s001.docx]

**APPENDIX A**

**QUESTIONNAIRE**

**VR Soccer Trainer**

*Test Subject Profile*

No: 年齢: サッカーの経験年数: 主なポジション:

A.ゴールキーパー B. ディフェンダー C. ミッドフィルダー D. フォワード

サッカーのレベル:

1. 初心者 B. アマチュア C. セミプロ D. プロ

以下の質問に対して、自分が最も近いと思った選択肢を選んでください。:

1. コンピューターで作られた世界の中で、私はそこにいる感じがした。

1 2 3 4 5 6 7

全くなし　　　 　　　　　　　　　　　　　　　 　非常に強い

1. 私は仮想世界に取り囲まれている気がした。

1 2 3 4 5 6 7

全くその通りでない　　　 　　　　　　　　　　　　　 　　全くその通り

1. 私はただ単に映像を見ているような気がした。

　　　　1 2 3 4 5 6 7

全くその通りでない　　　 　　　　　　　　　　　　　 　　全くその通り

1. 私は仮想空間にいる気がしなかった。

　　　　1 2 3 4 5 6 7

気がしなかった　　　 　　　　　　　　　　　　　 　　　　気がした。

1. 私は何かを外部から操作しているのではなく、仮想空間の中で振る舞っているような気がした。

　　　　1 2 3 4 5 6 7

全くその通りでない　　　 　　　　　　　　　　　　　 　　全くその通り

1. 私は仮想空間の中に居合わせているように感じた。

　　　　1 2 3 4 5 6 7

全くその通りでない　　　 　　　　　　　　　　　　　 　　全くその通り

1. あなたは、仮想世界を通って移動して行く間に、周りの現実世界をどのくらい意識していましたか。（例えば物音、室温、他の人間など）

　　　　1 2 3 4 5 6 7

極度に意識した　　　 　　　普通に意識した　　 　　　　意識しなかった

1. 私は、現実環境をもはや意識しなかった。

　　　　1 2 3 4 5 6 7

全くその通りでない　　　 　　　　　　　　　　　　 　　全くその通り

1. 私は未だ現実環境を注意していた。

　　　　1 2 3 4 5 6 7

全くその通りでない　　　 　　　　　　　　　　　　 　　全くその通り

1. 私は仮想世界によって完全に魅了されていた。

　　　　1 2 3 4 5 6 7

全くその通りでない　　　 　　　　　　　　　　　　 　　全くその通り

1. あなたは、仮想世界がどのくらい現実のように見えましたか。

　　　　1 2 3 4 5 6 7

全くの現実のよう　　　 　　　　　　　　　　　 　全くの現実のようでない

1. あなたの仮想環境の経験は、あなたの現実環境の経験とどのくらい似ていましたか

　　　　1 2 3 4 5 6 7

全くの似ていなかった　　　 　多少似ていた　　　　　　　全く似ていた

1. あなたには仮想世界がどのくらい現実のように見えましたか？

　　　　1 2 3 4 5 6 7

想像された世界のよう　　　 　　　　　　　　　　　　現実世界と区別ができない

1. 私には現実世界よりも仮想世界の方がより現実に見えた。

　　　　1 2 3 4 5 6 7

全くその通りでない　　　 　　　　　　　　　　　 　　全くその通り

コメントや提案があればお書きください。
